# Adapting the Thinking Healthy Programme for Perinatal Depression: A Culturally Tailored Approach in Three Central African Countries

**DOI:** 10.1192/j.eurpsy.2025.386

**Published:** 2025-08-26

**Authors:** E. Dozio, V. Wamba, I. Pueugueu

**Affiliations:** 1 Action contre la Faim, Paris, France; 2 Action contre la Faim, Goma, Congo, The Democratic Republic of the; 3 Action contre la Faim, Bangui, Central African Republic

## Abstract

**Introduction:**

In humanitarian settings, populations face extreme adversity, and women in the perinatal period are particularly vulnerable, often at heightened risk of depression. This impacts not only their mental health but also their ability to care for themselves and their newborns, presenting a serious challenge for maternal and nfant well-being.

**Objectives:**

This project aimed to reduce the risk of perinatal depression while strengthening infant care practices and parenting skills, ensuring that mothers, despite living in distressing and hostile environments, can be in the best possible state of mind to care for their babies.

**Methods:**

As part of Action contre la Faim’s psychosocial support projects, we adapted the WHO’s “Thinking Healthy” (TH) protocol specifically for low- and middle-income countries (LAMIC), focusing on cultural sensitivity and the unique challenges of the intervention areas. The standard manual was condensed into three sessions, with additional cultural adaptations and the inclusion of two projective sessions (protolanguage approach) to allow women more freedom to express their specific challenges. The protocol was delivered to groups of up to eight women, separated based on whether they were pregnant or breastfeeding to better target their unique needs. Due to logistical and security constraints, the TH protocol required further adaptation to fit each context’s specific limitations.

**Results:**

Over the past three years, the adapted TH protocol has been implemented in three countries across Central Africa, including both humanitarian crisis zones and more stable developmental settings. The programme reached approximately 5,000 preganant women, mothers, and their babies. It was delivered not only in healthcare centres but also directly in communities and internally displaced person (IDP) camps, providing wider access. Results demonstrated significant reductions in psychological distress and depressive symptoms, with improved mother-infant interactions. The programme also helped train healthcare workers, including midwives, enhancing local capacity for long-term support. Quantitative and qualitative results, along with details of cultural adaptations, will be presented.

**Conclusions:**

The adaptation of the Thinking Healthy protocol for low-resource, high-stress environments proved to be an effective and scalable approach for addressing perinatal depression. By tailoring the intervention to fit the cultural and logistical realities of Central Africa, we were able to provide meaningful support to thousands of families. The programme not only reduced depressive symptoms but also fostered stronger maternal-infant bonds and built local healthcare capacity. This model can serve as a reference for implementing mental health interventions in similar contexts globally.

**Disclosure of Interest:**

None Declared

